# Risk factors for recurrent IgA nephropathy after renal transplantation: A meta-analysis

**DOI:** 10.17305/bjbms.2022.8369

**Published:** 2023-05-01

**Authors:** Jiang Bai, Qiong Wu, Jing Chen, Zhifang Zheng, Jiarong Chang, Liangliang Wang, Yun Zhou, Qiang Guo

**Affiliations:** 1 Second Clinical Medical College, Shanxi Medical University, Taiyuan, Shanxi, China; 2 Shanxi Medical University, Taiyuan, Shanxi, China; 3 Department of Nephrology, Shanxi Provincial People’s Hospital (Fifth Hospital) of Shanxi Medical University, Taiyuan, Shanxi, China; 4 Department of Urology, The Second Hospital of Shanxi Medical University, Taiyuan, Shanxi, China

**Keywords:** Meta-analysis, IgA nephropathy (IgAN), kidney transplantation, risk factors

## Abstract

Recurrent glomerulonephritis after renal transplantation is the third most common cause of allograft loss, the most frequent of which is associated with IgA nephropathy (IgAN). This study aims to provide a systematic review of the risk factors associated with recurrent IgAN after renal transplantation. We searched English and Chinese databases, including PubMed, Embase, Web of Science, CNKI, and others, and included all case-control studies involving risk factors for recurrent IgAN after renal transplantation from the databases’ establishment to March 2022. Data were analyzed using the Stata 12.0. A total of 20 case-control studies were included in the meta-analysis, with 542 patients with recurrent IgAN and 1385 patients without recurrent IgAN. The results showed that donor age (standardized mean difference [SMD] −0.13 [95% confidence interval (CI) −0.26, −0.001]; *P* ═ 0.048), patient age at transplantation (SMD −0.41 [95% CI −0.53, −0.29]; *P* < 0.001), time from diagnosis to end-stage renal disease (SMD −0.42 [95% CI −0.74, −0.10]; *P* ═ 0.010), previous transplantation (odds ratio [OR] 1.73 [95% CI 1.06, 2.81]; *P* ═ 0.027), living donor (OR 1.86 [95% CI 1.34, 2.58]; *P* < 0.001), related donor (OR 2.64, [95% CI 1.84, 3.79]; *P* < 0.001), tacrolimus use (OR 0.71 [95% CI 0.52, 0.98]; *P* ═ 0.035), basiliximab use (OR 0.39 [95% CI 0.27, 0.55]; *P* < 0.001), proteinuria (SMD 0.42 [95% CI 0.13, 0.71]; *P* ═ 0.005), and serum IgA level (SMD 0.48 [95% CI 0.27, 0.69]; *P* < 0.001) were associated with recurrent IgAN after renal transplantation. In general, tacrolimus and basiliximab use were the protective factors against recurrent IgAN after renal transplantation, whereas donor age, patient age at transplantation, time from diagnosis to end-stage renal disease, previous transplantation, living donor, related donor, proteinuria, and serum IgA level were the risk factors for recurrent IgAN after renal transplantation. Clinical decision making should warrant further consideration of these risk factors.

## Introduction

End-stage renal disease (ESRD) is a worldwide public health concern. It imposes a significant burden on patients and often has a poor prognosis. Primary glomerulonephritis with IgA nephropathy (IgAN) is the leading cause of ESRD [1]. Currently, treatment modalities for ESRD include hemodialysis, peritoneal dialysis, and renal transplantation. Studies show that kidney transplantation is the most cost-effective approach [2–5]. More than 80% of patients with IgAN are young and middle-aged patients with fewer underlying diseases, so they can usually be ideal candidates for renal transplantation. In fact, IgAN patients account for a high proportion (approximately 13%) of candidates for renal transplantation [6]. However, recurrent glomerulonephritis after renal transplantation is the third most common cause of allograft loss, the most frequent of which is associated with IgAN [6–11]. Some studies have shown that the proportion of IgAN recurrence ranges from 9% to 53% due to different follow-up times and biopsy protocols [12].

Research suggested that recurrent IgAN after renal transplantation might be associated with younger age at transplantation, living related donor, rapidly progressive course of the original disease [12, 13], higher levels of circulating galactose-deficient IgA1 (Gd-IgA1), and other factors [14], whereas some of the above factors remained controversial. Therefore, accurate identification of risk factors associated with recurrent IgAN after kidney transplantation is a key for selecting transplant candidates and might help improve the long-term survival rate of patients.

## Materials and methods

### Literature search strategy

We searched English and Chinese databases, including PubMed, Embase, Medline, Web of Science, Cochrane Library, CNKI, CBMdisc, Wanfang and Weipu (VIP), and included all case-control studies on risk factors for recurrent IgAN after kidney transplantation, from the databases ’ establishment to March 2022. The risk of bias and quality was assessed according to Newcastle–Ottawa Scale. The relevant papers were identified using Medical Subject Headings (MeSH) terms: “Glomerulonephritis,” “Glomerulonephritides,” “Kidney Scarring,” “Kidney Transplantation,” “Renal Transplantation,” “Scarring, Kidney” and other free words. The idiographic search strategy retrieval is shown in Supplementary Material 1. Simultaneously, manual search of the included literature was performed to eliminate duplicate literature. Recurrent IgAN was defined by mesangial IgA deposition on immunofluorescence staining of allograft biopsy when clinical features of kidney transplantation recipients were hematuria, increasing proteinuria, elevated serum IgA levels and allograft dysfunction (defined by a significant increase in serum creatinine).

**Table 1:** Characteristics and Newcastle–Ottawa Scale quality score of included studies

**Author**	**Year**	**NOS**	**Country**	**Recurrent IgAN (*n*)**	**Sample (*n*)**	**Period**	**Risk factors**
Okumi M et al.	2019	8	Japan	80	299	1995–2015	Donor age, living donor, related donor, previous transplantation, tacrolimus use, basiliximab use, serum IgA level
Martin-Penagos et al.	2019	8	Spain	14	35	1993—2015	Donor age, previous transplantation, tacrolimus use, basiliximab use, proteinuria
Jo et al.	2019	8	Korea	11	69	2011–2015	Previous transplantation, living donor, related donor, tacrolimus use
Di Vico et al.	2018	7	Italy	28	51	1995–2012	Donor age, age at transplantation, previous transplantation, living donor, tacrolimus use, basiliximab use, proteinuria
Garnier et al.	2018	8	France	14	67	2003–2013	Donor age, age at transplantation, previous transplantation, living donor, tacrolimus use, basiliximab use, proteinuria, serum IgA level
Temurhan et al.	2017	7	Turkey	18	41	NA	Donor age, age at transplantation, living donor, tacrolimus use
Avasare et al.	2017	8	USA	14	62	2001–2012	Age at transplantation, time from diagnosis to ESRD, living donor, proteinuria
Sato et al.	2013	7	Japan	70	184	1990–2005	Donor age, age at transplantation, living donor, related donor, tacrolimus use, basiliximab use
Moroni et al.	2013	8	Italy	42	190	1981–2010	Age at transplantation, living donor, tacrolimus use, basiliximab use
Lida et al.	2020	8	Spain	23	83	1992–2016	Proteinuria
Namba et al.	2004	7	Japan	21	27	1980–2001	Age at transplantation, living donor
Coppo et al.	2007	7	Italy	30	61	NA	Age at transplantation, related donor, proteinuria
Ponticelli et al.	2001	7	Italy	34	106	NA	Age at transplantation, living donor, proteinuria
Wang et al.	2001	7	China	14	48	1985–1998	Age at transplantation, time from diagnosis to ESRD, related donor, serum IgA level
Ortiz et al.	2012	8	Spain	21	65	2001–2010	Donor age, age at transplantation, related donor, tacrolimus use, proteinuria
Bumgardner et al.	1998	7	USA	15	54	NA	Age at transplantation, living donor, related donor
Park et al.	2021	7	Korea	13	27	2009–2016	Donor age, age at transplantation, living donor, related donor, tacrolimus use, basiliximab use, proteinuria
Moriyama et al.	2005	7	Japan	13	49	1992–1999	Donor age, age at transplantation, living donor, related donor, proteinuria, serum IgA level
Lionaki et al.	2021	8	Greece	23	96	NA	Donor age, age at transplantation, time from diagnosis to ESRD, living donor, related donor, tacrolimus use
Maixnerova et al.	2021	7	Czech Republic	44	313	1991–2017	Donor age, age at transplantation, previous transplantation, living donor

NA: Not available; NOS: Newcastle–Ottawa Scale; ESRD: End-stage renal disease; IgAN: IgA nephropathy.

### Literature selection and data extraction

All literature selection and necessary data extraction were performed by two independent reviewers (ZZ and JC). If there were disagreements, the reviewers discussed them, and a third researcher adjudicated them (QG, blinded to the authors and institute of studies). The following were the criteria for inclusion: 1) type of study: published case-control studies containing clinical data from the groups with recurrent IgAN and groups without recurrent IgAN; 2) the full text was available on the Internet; 3) exposure factors: risk factors for recurrent IgAN in kidney transplant recipients and outcome indicators. The following were the criteria for exclusion: 1) repeated publications; 2) insufficient full text, partial data, inconvertible data, or no control group; 3) no biopsy and patients replaced by number of grafts. During the screening process, obviously, ineligible literature was excluded first by reading titles and abstracts, and then the full text of literature that might meet the requirements was read to determine whether it met the inclusion criteria. The extracted data included literature title, publication time, first author, sample size, risk factors, number of cases in the group with recurrent IgAN and group without recurrent IgAN, etc.

### Ethical statement

Ethical approval was not required for this study in accordance with local/national guidelines. Written informed consent to participate in the study was not required in accordance with local/national guidelines. The protocol was registered on PROSPERO (CRD42022315448).

### Statistical analysis

We conducted data integration and analysis using Stata 12.0. We performed a meta-analysis of the risk factors that were included in more than two studies. Odds ratios (OR) and 95% confidence intervals (CI) were selected for dichotomous data on possible risk factors from included studies. Continuous data were analyzed using a standardized mean difference (SMD) and 95% CI. *I*^2^ was used to assess the heterogeneity of the included literature data. If *I^2^* < 50%, it was considered that there was no heterogeneity, and the fixed effect model was adopted. Otherwise, the random effect model was used. To examine publication bias, the funnel plot and Egger’s test were used. Tables 2 and 3 present the results of the risk factor analysis with publication bias. In the case of statistical heterogeneity, we performed the subgroup analysis to identify the sources of heterogeneity. Moreover, the sensitivity analysis was performed to evaluate the stability of the pooled results. The graphs were created using R 3.6.3.

**Figure 1.**
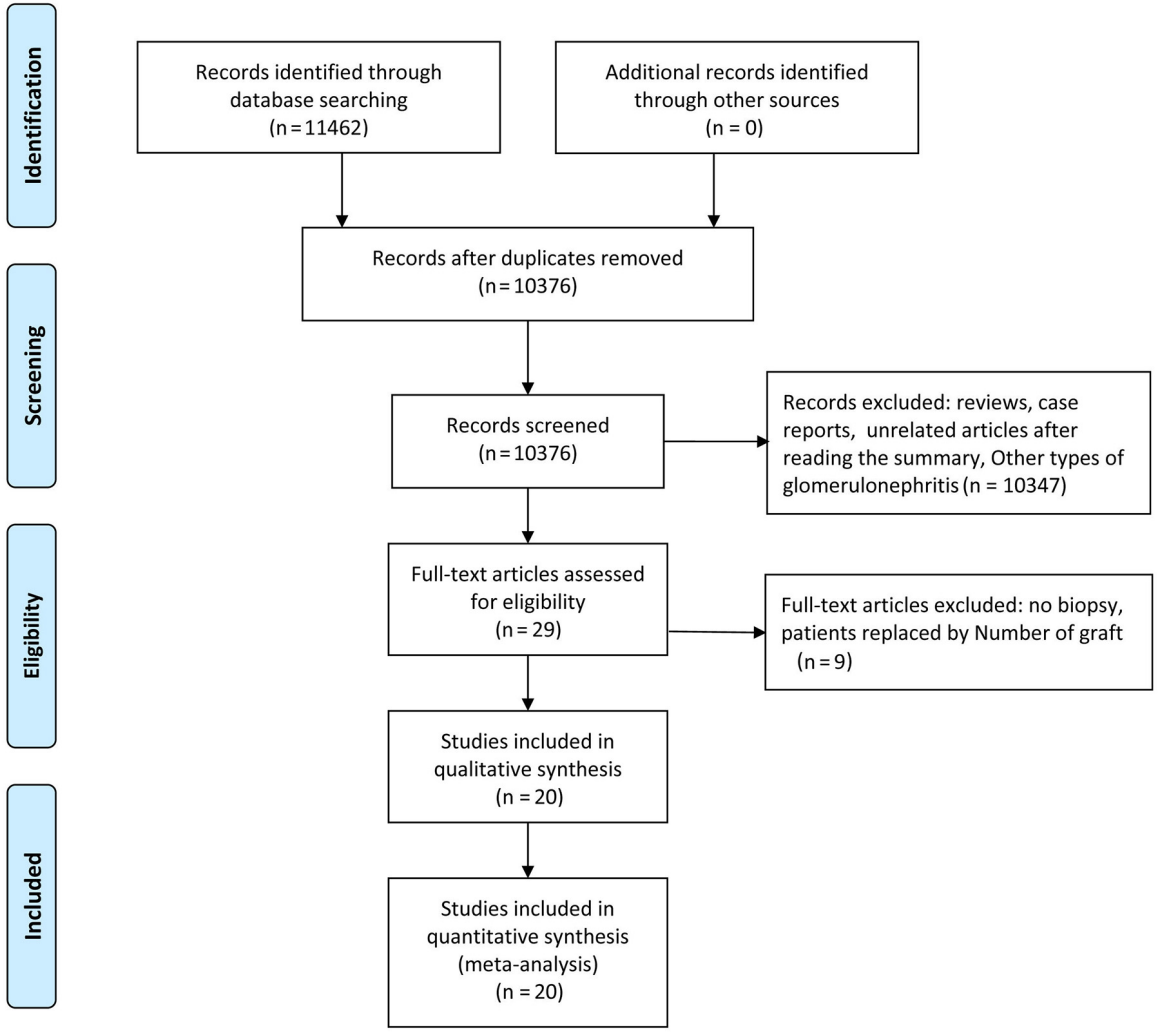
The process of the identification and inclusion of selected studies.

**Table 2:** Summary of meta-analysis results of non-modifiable factors for recurrent IgA nephropathy after renal transplantation

**Factors**	**No of studies**	**Total number of patients**	**Heterogeneity test**		**OR/SMD (95% CI)**	*P* **value**	**Egger’s test**	
			*P*	*I^2^*			*t*	*P*
Donor age	11	1227	0.067	42.3	−0.13 (−0.26, −0.001)	0.048	−1.40	0.196
Patient age at transplantation	16	1441	0.229	19.6	−0.41 (−0.53, −0.29)	<0.001	0.44	0.663
Time from diagnosis to ESRD	3	206	0.764	0	−0.42 (−0.74, −0.10)	0.01	−3.47	0.179
Previous transplantation	6	834	0.205	30.7	1.73 (1.06, 2.81)	0.027	0.36	0.734
Living donor	15	1635	0.641	0	1.86 (1.34, 2.58)	<0.001	0.98	0.344
Related donor	10	952	0.879	0	2.64 (1.84, 3.79)	<0.001	−0.99	0.350

OR: Odds ratio; SMD: Standardized mean difference; CI: Confidence interval; ESRD: End-stage renal disease.

**Table 3:** Summary of meta-analysis results of modifiable factors for recurrent IgA nephropathy after renal transplantation

**Factors**	**No of studies**	**Total number of patients**	**Heterogeneity test**		**OR/SMD (95% CI)**	*P* **value**	**Egger’s test**	
			*P*	*I^2^*			*t*	*P*
Tacrolimus use	11	1124	0.041	47.2	0.71 (0.52, 0.98)	0.035	−0.32	0.758
Basiliximab use	7	853	0.078	47.2	0.39 (0.27, 0.55)	<0.001	0.90	0.408
Proteinuria	10	606	0.005	62.1	0.42 (0.13, 0.71)	0.005	2.41	0.043
Serum IgA level	4	463	0.582	0	0.48 (0.27, 0.69)	<0.001	10.67	0.009

OR: Odds ratio; SMD: Standardized mean difference; CI: Confidence interval.

## Results

### Basic characteristics of the included studies and results of quality assessment

A total of 1927 patients were included in this study, including 542 patients with recurrent IgAN and 1385 patients without recurrent IgAN. Table 1 shows the basic information and characteristics of the 20 articles included and the results of quality assessment according to the Newcastle–Ottawa Scale. An overview of the results and process of literature screening is shown in Figure 1. The results of modifiable and non-modifiable risk factors are presented in Tables 2 and 3.

### Non-modifiable factors

#### Donor age

**Figure 2.**
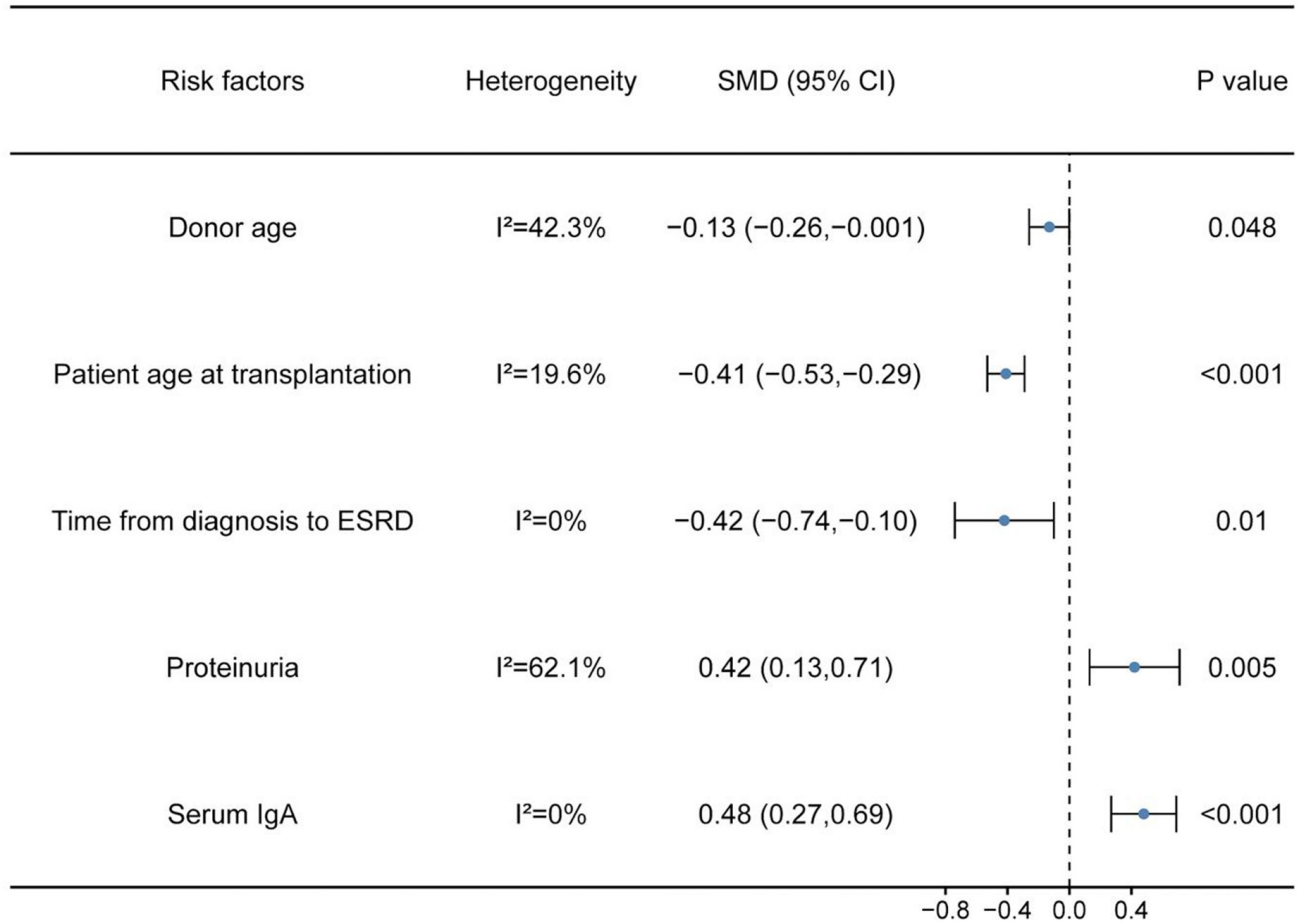
**SMD and the corresponding 95% CIs for risk factors for recurrent IgAN.** Young donor age, age at transplantation, short time from IgAN diagnosis to ESRD, high level of proteinuria, and serum IgA level were associated with an increased risk for recurrent IgAN after renal transplantation. SMD: Standardized mean difference; CI: Confidence interval; ESRD: End-stage renal disease; IgAN: IgA nephropathy.

Eleven studies [15–25] were included, with 338 patients in the recurrent IgAN group and 889 patients in the group without recurrent IgAN. The heterogeneity test showed *I^2^* ═ 42.3%, *P* ═ 0.067 and was analyzed using a fixed effect model. The comparative difference between the two groups was statistically significant (SMD −0.13 [95% CI −0.26, −0.001]; *P* ═ 0.048]. Young donor age was associated with an increased risk for recurrent IgAN after renal transplantation (Figure 2).

**Figure 3.**
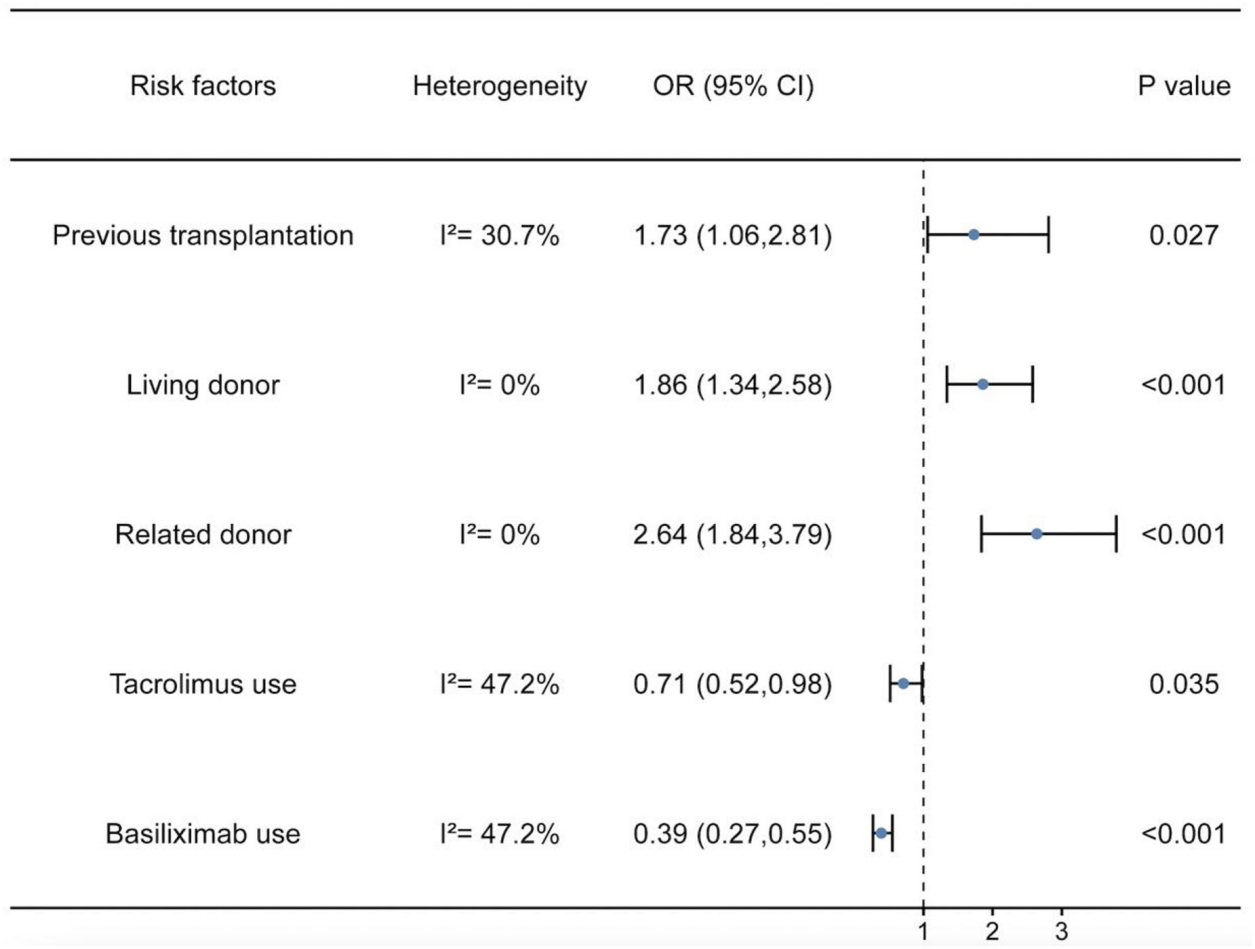
**Odds ratios and the corresponding 95% CI for risk factors for recurrent IgAN.** Previous transplantation, living donor, and related donor were risk factors for recurrent IgAN after renal transplantation. Tacrolimus and basiliximab use were protective factors against recurrent IgAN after renal transplantation. OR: Odds ratio; CI: Confidence interval; IgAN: IgA nephropathy.

#### Patient age at transplantation

**Figure 4.**
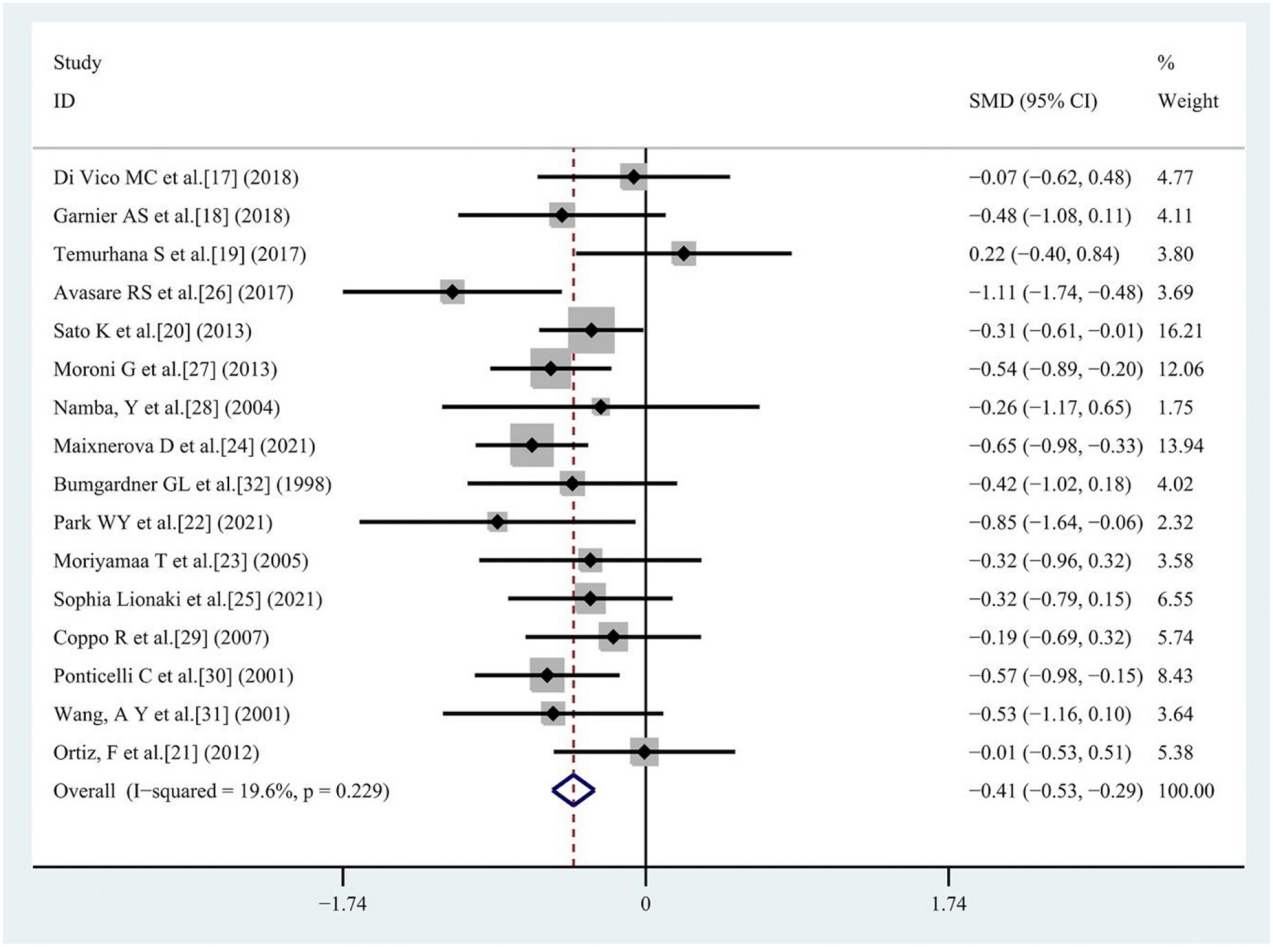
**Forest plot of patient young age at transplantation as a risk factor.** SMD: Standardized mean difference; CI: Confidence interval.

Sixteen studies [17–32] were included, with 414 patients in the recurrent IgAN group and 1027 patients in the group without recurrent IgAN. The heterogeneity test showed *I^2^* ═ 19.6%, *P* ═ 0.229 and was analyzed using a fixed effect model. The comparative difference between the two groups was statistically significant (SMD −0.41 [95% CI −0.53, −0.29]; *P* < 0.001). Young patient age at transplantation was associated with an increased risk for recurrent IgAN after renal transplantation (Figure 4).

#### Time from diagnosis to ESRD

Three studies [25, 26, 31] were included, with 51 patients in the recurrent IgAN group and 155 patients in the group without recurrent IgAN. The heterogeneity test showed *I^2^* ═ 0%, *P* ═ 0.764, and was analyzed using a fixed effect model. The comparative difference between the two groups was statistically significant (SMD −0.42 [95% CI −0.74, −0.1]; *P* ═ 0.010). Short time from diagnosis to ESRD was associated with an increased risk for recurrent IgAN after renal transplantation (Figure 2).

#### Previous transplantation

Six studies [15, 18, 24] were included, with 191 patients in the recurrent IgAN group and 643 patients in the group without recurrent IgAN. The heterogeneity test showed *I^2^* ═ 30.7%, *P* ═ 0.205, and was analyzed using a fixed effect model. The comparative difference between the two groups was statistically significant (OR 1.73 [95% CI 1.06, 2.81]; *P* ═ 0.027). The previous transplantation was a risk factor for recurrent IgAN after renal transplantation (Figure 3).

#### Living donor

**Figure 5.**
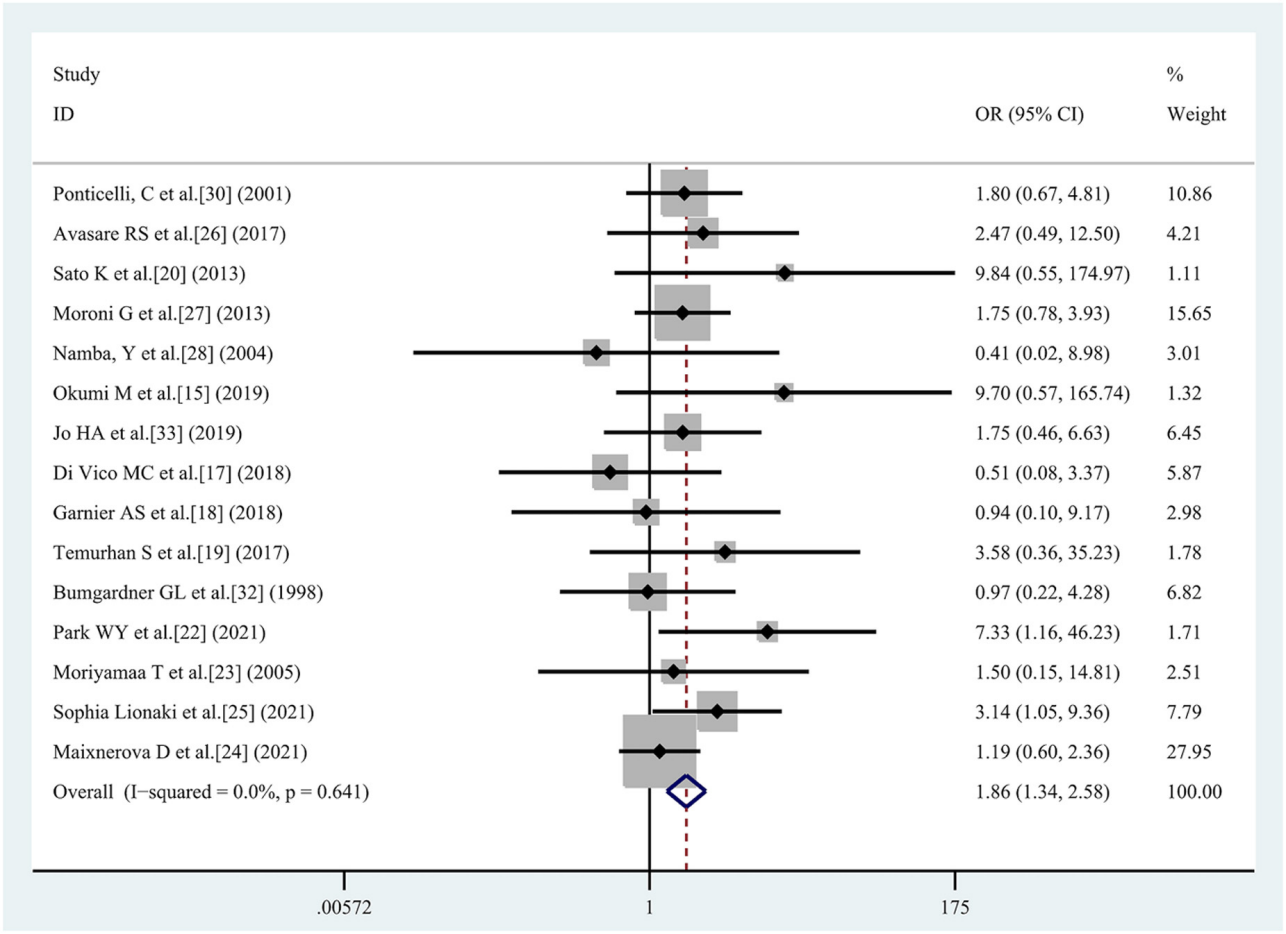
**Forest plot of living donor as a risk factor.** OR: Odds ratio; CI: Confidence interval.

Fifteen studies [15, 17–20, 22–28, 30, 32, 33] were included, with 440 patients in the recurrent IgAN group and 1195 patients in the group without recurrent IgAN. The heterogeneity test showed *I^2^* ═ 0%, *P* ═ 0.641, and was analyzed using a fixed effect model. The comparative difference between the two groups was statistically significant (OR 1.86 [95% CI 1.34, 2.58]; *P* < 0.001). The living donor was a risk factor for recurrent IgAN after renal transplantation (Figure 5).

#### Related donor

Ten studies [15, 20–23, 25, 29, 31–33] were included, with 290 patients in the recurrent IgAN group and 662 patients in the group without recurrent IgAN. The heterogeneity test showed *I^2^* ═ 0%, *P* ═ 0.879, and was analyzed using a fixed effect model. The comparative difference between the two groups was statistically significant (OR 2.64 [95% CI 1.84, 3.79]; *P* < 0.001). The related donor was a risk factor for recurrent IgAN after renal transplantation (Figure 3).

### Modifiable risk factors

#### Tacrolimus use

Eleven studies [15–22, 25, 27, 33] were included, with 334 patients in the recurrent IgAN group and 790 patients in the group without recurrent IgAN. The heterogeneity test showed *I^2^* ═ 47.2%, *P* ═ 0.041, and was analyzed using a fixed effect model. The comparative difference between the two groups was statistically significant (OR 0.71 [95% CI 0.52, 0.98]; *P* ═ 0.035). The tacrolimus use was a protective factor for recurrent IgAN after renal transplantation (Figure 3).

#### Basiliximab use

Seven studies [15–18, 20, 22, 27] were included, with 261 patients in the recurrent IgAN group and 592 patients in the group without recurrent IgAN. The heterogeneity test showed *I^2^* ═ 47.2%, *P* ═ 0.078, and was analyzed using a fixed effect model. The comparative difference between the two groups was statistically significant (OR 0.39 [95% CI 0.27, 0.55]; *P* < 0.001). The basiliximab use was a protective factor for recurrent IgAN after renal transplantation (Figure 3).

#### Proteinuria

Ten studies [16–18, 21–23, 26, 29, 30, 34] were included, with 204 patients in the recurrent IgAN group and 402 patients in the group without recurrent IgAN. The heterogeneity test showed *I^2^* ═ 62.1%, *P* ═ 0.005, and was analyzed using a random effect model. The comparative difference between the two groups was statistically significant (SMD 0.42 [95% CI 0.13, 0.71]; *P* ═ 0.005). The high level of proteinuria was associated with an increased risk for recurrent IgAN after renal transplantation (Figure 2).

We performed a subgroup analysis to identify the sources of heterogeneity. Subgroup analysis revealed that there were no differences in the proteinuria at the time of six months (*I^2^* ═ 68.3%, SMD 0.33 [95% CI −0.31, 0.97]; *P* ═ 0.312), one year (*I^2^* ═ 21.1%, SMD 0.30 [95% CI −0.09, 0.68]; *P* ═ 0.130) and at biopsy (*I^2^* ═ 0%, SMD 0.21 [95% CI −0.13, 0.55]; *P* ═ 0.229) after transplantation (Supplementary Material 2).

#### Serum IgA level

Four studies [15, 18, 31, 33] were included, with 121 patients in the recurrent IgAN group and 342 patients in the group without recurrent IgAN. The heterogeneity test showed *I^2^* ═ 0%, *P* ═ 0.582, and was analyzed using a fixed effect model. The comparative difference between the two groups was statistically significant (SMD 0.48 [95% CI 0.27, 0.69]; *P* < 0.001). The high level of serum IgA was associated with an increased risk for recurrent IgAN after kidney transplantation (Figure 2). Subgroup analysis revealed that there was the high level of serum IgA in the recurrent IgAN group at the time of six months (SMD 0.68 [95% CI 0.08, 1.28]; *P* ═ 0.027), at three years (SMD 0.43 [95% CI 0.19, 0.67]; *P* < 0.001), and at the time of diagnosis of recurrent IgAN (SMD 0.65 [95% CI 0.02, 1.29]; *P* ═ 0.045) after transplantation (Supplementary Material 2).

**Figure 6.**
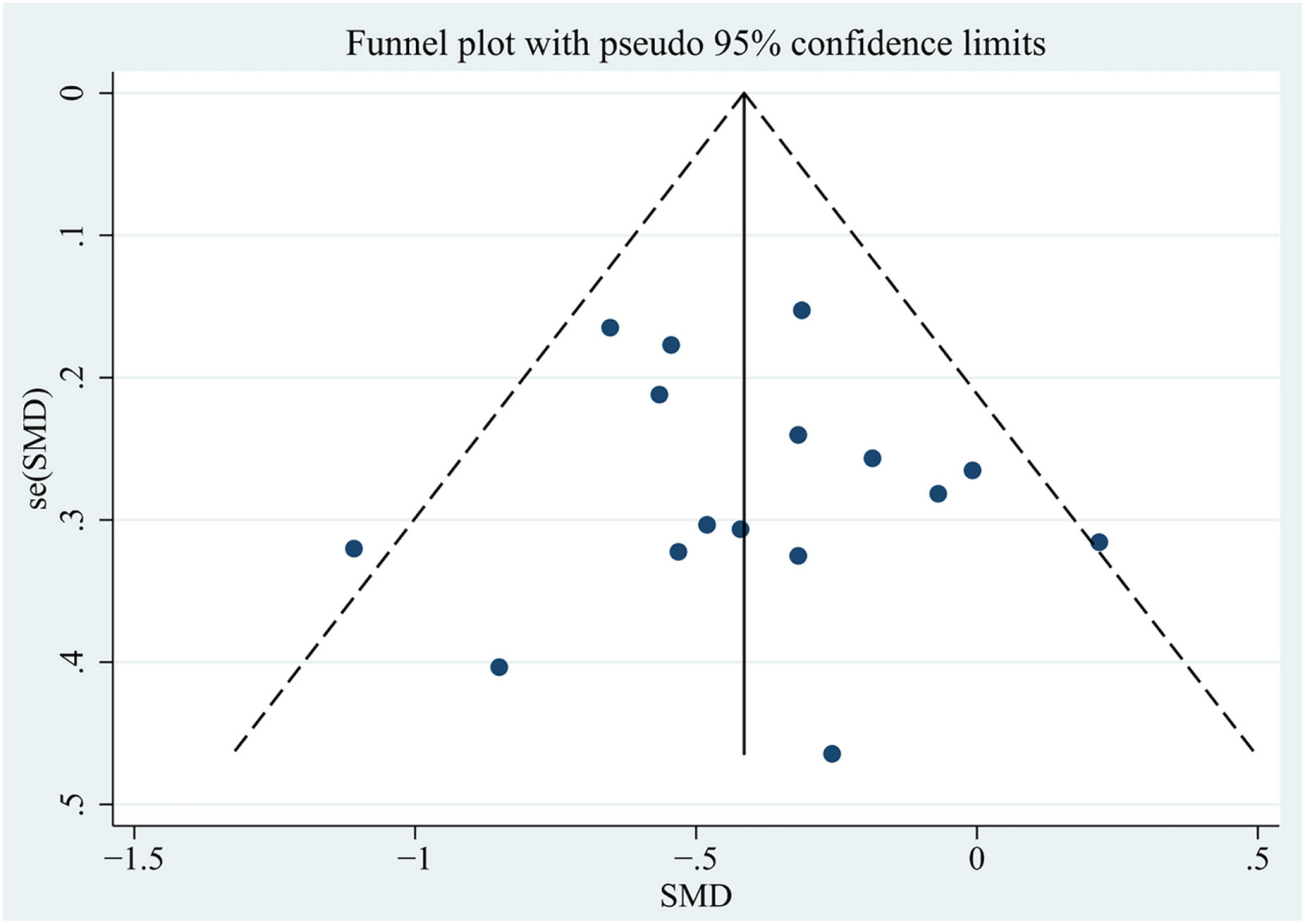
**Funnel chart results for young patient age at transplantation as a risk factor.** SMD: Standardized mean difference; SE: Standard error.

**Figure 7.**
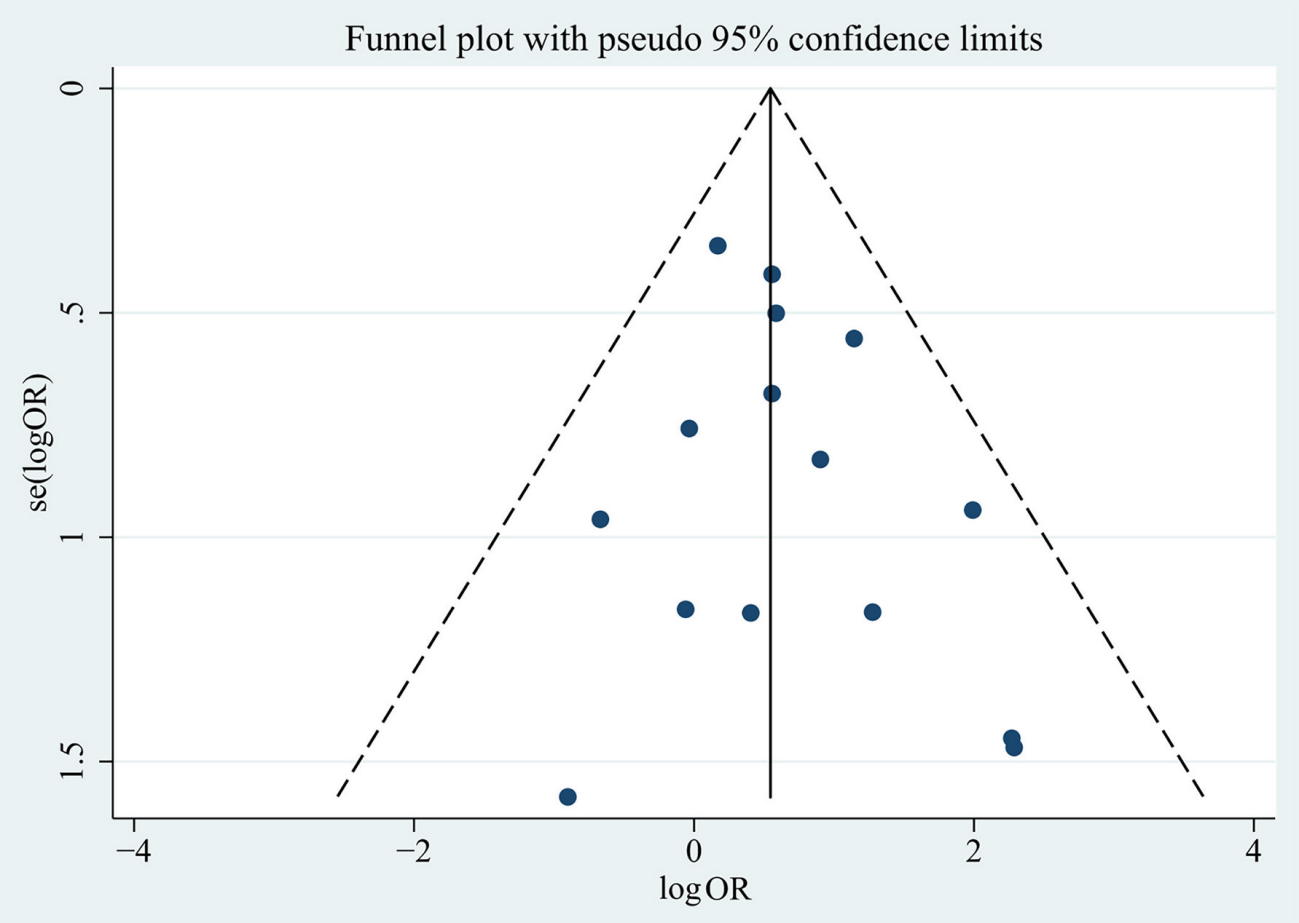
**Funnel chart results for living donor as a risk factor.** OR: Odds ratio.

**Figure 8.**
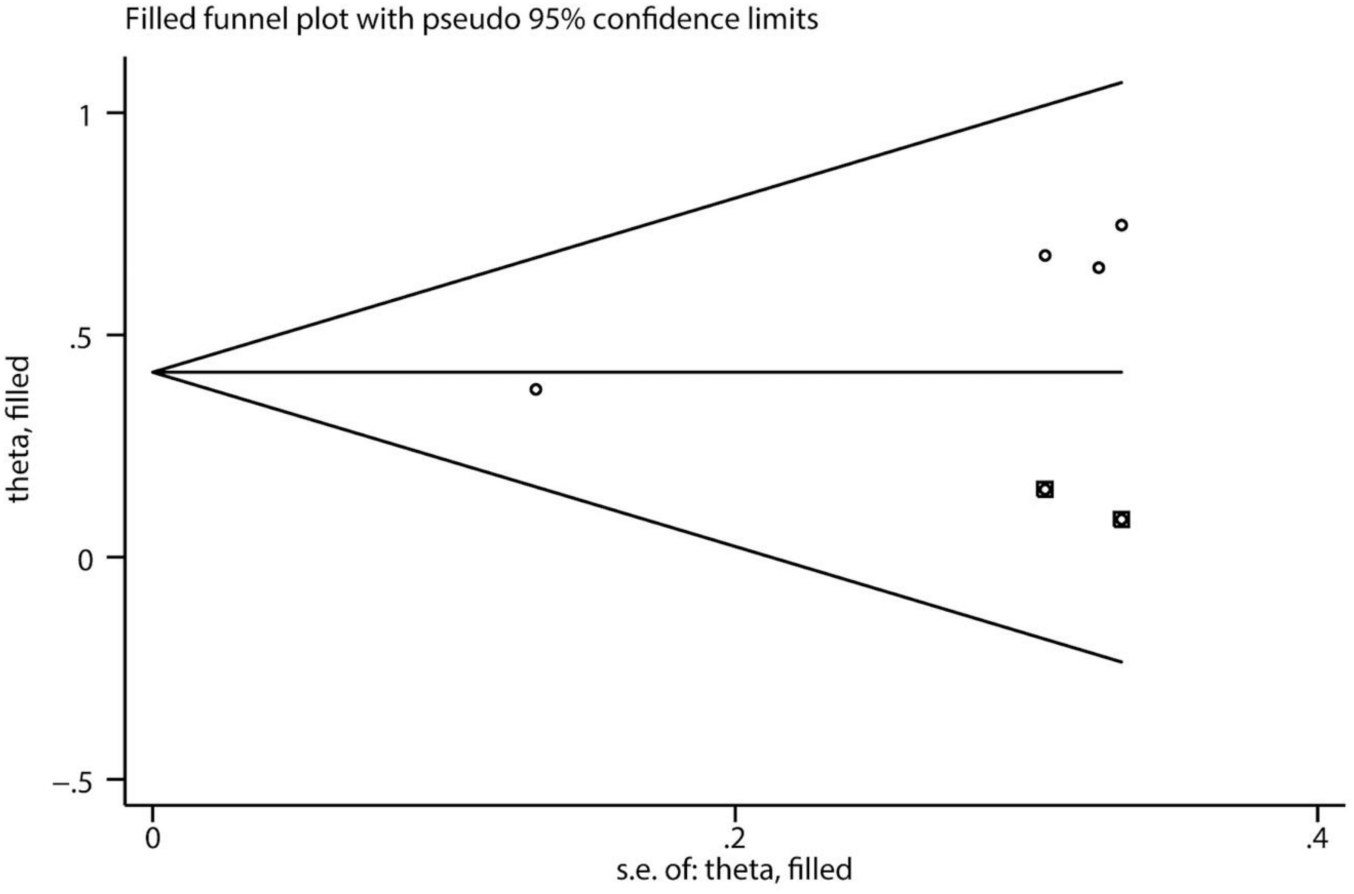
**The results for serum IgA in the trim-and-fill analysis.** SE: Standard error.

**Figure 9.**
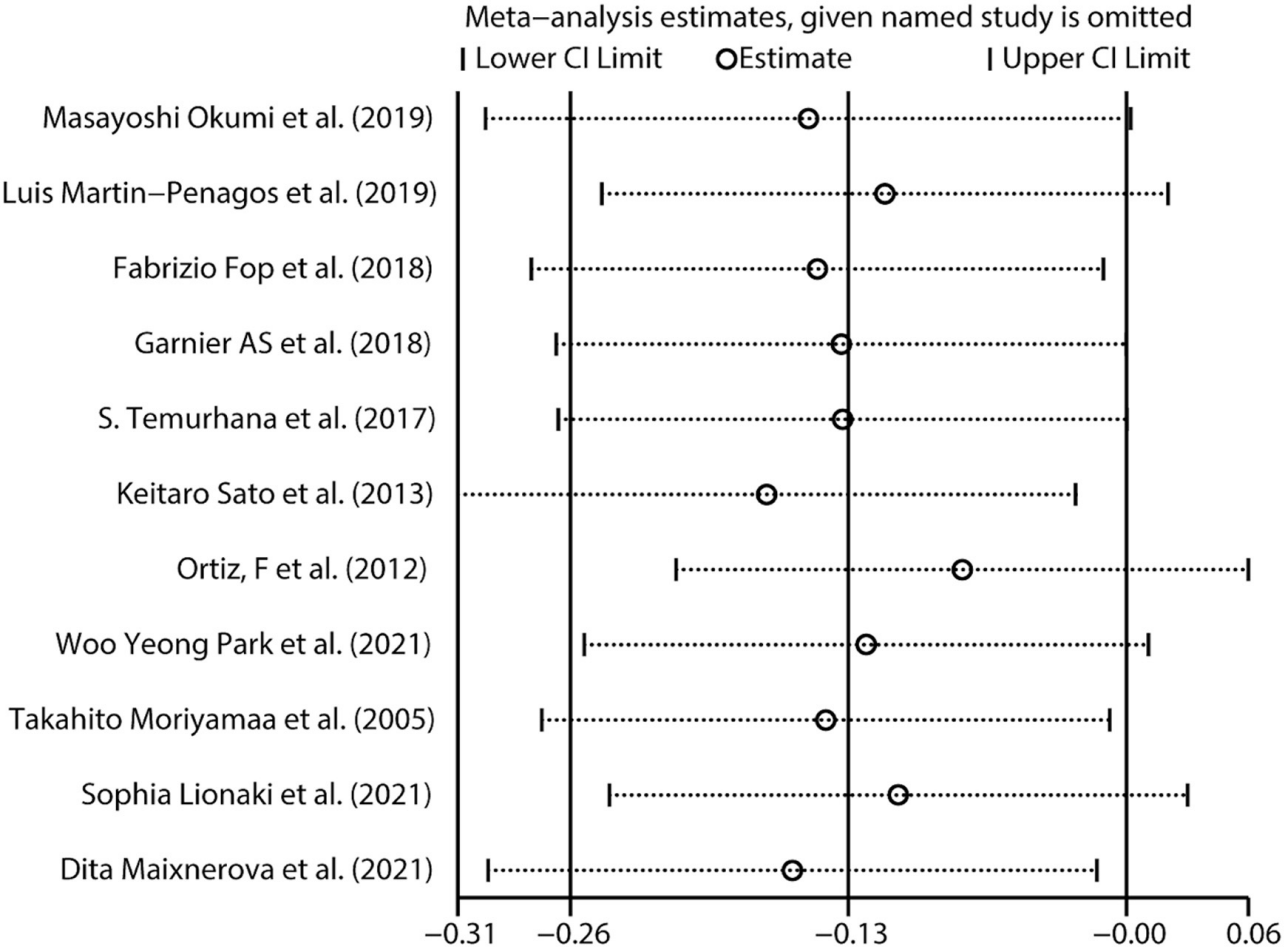
**The results for donor age in the sensitivity analysis.** CI: Confidence interval.

### Results of publication bias assessment

Using the Egger’s test and funnel plots, we assessed publication bias (Figures 6 and 7, Supplementary Material 2). Furthermore, Tables 2 and 3 present the results of the risk factor analysis with publication bias. As a result, donor age, patient age at transplantation, time from diagnosis to ESRD, previous transplantation, living donor, related donor, and tacrolimus and basiliximab use did not demonstrate publication bias. The Egger’s test of proteinuria (*t* ═ 2.41, *P* ═ 0.043) and serum IgA (*t* ═ 10.67, *P* ═ 0.009) indicated the existence of publication bias. The trim-and-fill analysis was used, and no potential “missing studies” were found in proteinuria. There were two potential “missing studies” found in serum IgA in the trim-and-fill analysis. The adjustment for publication bias had no obvious impact on the pooled estimate and the results of proteinuria (adjusted pooled SMD 0.42 [95% CI 0.13–0.71]; *P* ═ 0.005) and serum IgA (adjusted pooled SMD 0.42 [95% CI 0.23–0.61]; *P* < 0.001) remained stable (Figure 8, Supplementary Material 3). Sensitivity analysis revealed that donor age, time from diagnosis to ESRD, previous transplantation, and tacrolimus use were unstable. These four factors should be interpreted with caution (Figure 9, Supplementary Material 3).

## Discussion

In 1975, Berger et al. [35] first described the recurrence of IgAN in renal transplantation. In 1994, Odum et al. [36] reported up to 30% of graft loss rate secondary to recurrent IgAN in renal transplantation. Transplant engraftment after IgAN recurrence was significantly lower than in the group without recurrent IgAN. Therefore, research has attempted to identify relevant risk factors for IgAN recurrence to guide the clinical management of recurrent IgAN. Here, we provided a systematic review of the literature concerning several potential risk factors, that might help to classify the likelihood of renal transplantation patients developing recurrent IgAN. Specifically, donor age, patient age at transplantation, time from diagnosis to ESRD, previous transplantation, living donor, related donor, tacrolimus use, basiliximab use, proteinuria, and serum IgA level were included in the meta-analysis.

The present meta-analysis unraveled several organ donor-related risk factors for recurrent IgAN in renal transplant patients. Published studies showed the inconsistent results on whether donor age is a risk factor for IgAN recurrence following kidney donation. Whereas Lionaki et al. [25] reported that donor age was not associated with recurrent IgAN, other research groups demonstrated a link between donor age and the onset of recurrent IgAN [21]. Our meta-analysis results suggested that patients with recurrent IgAN were more likely to have received an organ from a young donor, than the patients without recurrent IgAN. However, the sensitivity analysis revealed that the results were unstable. The influence of donor–patient familial relationships on the incidence of recurrent IgAN is still debatable. Han et al. [37] found that donation from a living related donor was associated with a higher risk of the recurrence of IgAN (*P* < 0.05) compared to patients without recurrent IgAN. On the contrary, Maixnerova et al. [24] found that the living donor was not related to the recurrent IgAN. Our study showed that living donor transplantation increased the risk of recurrence of IgAN after renal transplantation, especially the living related donor (*P* < 0.05). Most living donor kidneys came from relatives, which led to a higher recurrence of IgAN compared to the deceased donor. On the one hand, in living related transplants, there is a higher degree of HLA matching between donors and recipients, which might be associated with the lower immunosupresion. On the other hand, IgAN has the phenomenon of familial aggregation, and genetic factors play a key role in the development of familial IgAN [38]. Therefore, the recurrence of familial IgAN was likely to occur when same-family relatives were selected as living donor kidney sources. Notably, previous studies showed that although transplantation from living related donors caused a higher recurrence of IgAN, it did not significantly increase the risk of graft loss [39].

We further identified post-transplantation recurrent IgAN risk factors associated with kidney recipients. First, consistent with previous studies, we observed that younger age at transplantation was associated with the group with recurrent IgAN compared to the group without recurrent IgAN. In a study concerning risk factors for the recurrent glomerulonephritis after renal transplantation, Allen et al. [13] found that recipient age was an independent risk factor for recurrent IgAN (*P* < 0.001). It could be explained that the stronger immune system of young patients, compared to the older ones, might lead to increased deposition of immune complexes. In clinical practice, immunosuppressant doses are calculated according to patients’ body weight, rarely accounting for age. Therefore, it is possible that the drug doses currently administered to younger patients might not be enough to achieve adequate immunosuppression, thus leading to a higher recurrence of IgAN. Moreover, the longer follow-up periods, which were observed in younger patients, might explain the higher diagnostic frequency of recurrent IgAN in this group, compared to the older patients [37].

In addition to previous factors, our results suggested that the time between the diagnosis of IgAN and the development of ESRD affected the recurrence of IgAN. Consistent with the previous studies, we observed a shorter time between diagnosis and ESRD onset in patients with recurrent IgAN patients than in the patients without recurrent IgAN. However, the sensitivity analysis revealed that the result was unstable; this might be due to the small number of included studies (only three). This meant that a greater risk of an IgAN recurrence was related to an initially progressing condition before transplantation. In clinical practice, the shorter the time from the diagnosis of IgAN to ESRD, the stronger the patient’s immune system, the more immune complexes will be deposited, and sufficient immunosuppression cannot be achieved, resulting in a higher recurrence rate of IgAN.

Analyses of a large sample size revealed that patients who were subjected to previous transplantation were at increased risk of IgAN recurrence [7]. Other studies, however, reported no association between previous transplantation and higher risk of recurrence [17, 24]. In the present systematic review, six studies reported previous transplantation as a risk factor for recurrent IgAN and this result was unstable. We hypothesized that the results might be biased due to limited sample size and inaccurate incidence estimates, resulting in wide confidence ranges in the studies with small samples. Previous studies showed that renal re-transplantation after the first graft failure was associated with higher survival benefit compared to dialysis [40, 41]. Additionally, no significant differences were observed in patient survival when compared to patients undergoing first renal transplantation [42–45]. Therefore, re-transplantation remains a suitable option for patients with initial graft loss due to IgAN recurrence.

When hematuria and/or proteinuria occurred, the diagnosis of recurrent IgAN might be obtained by indication biopsy [17]. However, Ortiz et al. [21] found that 52% of recurrent IgAN cases diagnosed by protocol biopsy were not accompanied by proteinuria or hematuria. Therefore, we reviewed proteinuria levels after transplantation. In the study from Coppo et al. [29], the average urinary protein excretion in patients with recurrent IgAN was significantly higher than in the control group (*P* ═ 0.002). Wang et al. [31] also confirmed that patients with graft dysfunction had more serious trend of proteinuria. Similarly, our meta-analysis revealed a higher proteinuria level after renal transplantation in patients with recurrent IgAN than in patients without recurrent IgAN. However, a subgroup analysis revealed that there was no difference in the proteinuria at six months, one year, and at biopsy after transplantation, which was consistent with Ortiz et al. Notably, the level of proteinuria before transplantation is also important and there is a widely held belief among clinicians that a more severe disease in patients was related to a higher risk of recurrence. This meant that the level of proteinuria pre-transplantation might be crucial in predicting recurrence in IgAN patients. In addition, whether proteinuria is a cause or a consequence of recurrent IgAN remains to be clarified. Nevertheless, strict control of proteinuria was beneficial for the prognosis of recurrent IgAN after renal transplantation [46].

The etiology of IgAN remains unclear, with the four-hit hypothesis being the most widely accepted theory on its pathophysiology. Unknown upstream mechanisms promote Gd-IgA1 production, which polymerizes and forms immunological complexes with autoantibodies. Cumulative IgA1 deposition stimulates mesangium growth and the release of several cytokines, chemokines, and extracellular matrix substances [47]. It was confirmed that Gd-IgA1 and serum IgA level in pre-transplantation served as biomarkers to predict IgAN recurrence [48]. Berthelot et al. [49] reported that lower levels of IgA–sCD89 immune complexes and higher levels of Gd-IgA1 or IgG–Gd-IgA1 complexes in pre-transplantation might indicate a higher risk of recurrence following transplantation. They discovered that sCD89 deposited in the mesangium, suggesting that sCD89–IgA complexes might play a role in the pathophysiology of IgAN recurrence. However, its detection was not widely implemented due to associated high costs and technical limitations. Instead, we advocated that serum IgA levels were potentially predictive of recurrent IgAN after renal transplantation. Our meta-analysis revealed an increase in serum IgA levels in patients with recurrent IgAN. Garnier et al. [18] found that kidney transplant recipients diagnosed with IgAN had higher levels of serum IgA, compared to patients with other nephropathies (*P* < 0.05). This was especially true for patients whose serum IgA levels at month 6 post-transplant were more than 222.5 mg/dL. Therefore, we proposed that high level of serum IgA was a risk factor for IgAN recurrence.

With the continuous development and use of immunosuppressive agents, the short-term engraftment rate of transplanted kidneys has been improved. However, the long-term survival rate of transplanted kidneys is still low, with the role of immunosuppressive therapy in the onset of recurrent IgAN yet to be clarified. Our study revealed that among immunosuppressant drugs, basiliximab and tacrolimus were protective against recurrent IgAN.

Basiliximab is one of the most commonly used interleukin 2 receptor antagonists (IL-2RA), widely used in immune induction therapy after renal transplantation [50]. Basiliximab targets activated T lymphocytes CD25 antigen, thus blocking the binding of IL-2. This leads to cell cycle arrest in G0 or G1 phase, thus inhibiting T cell proliferation [51]. T cells play a key role in immune response after renal transplantation, mediating cellular rejection [52]. In this context, granular complement deposition was a common pathological manifestation that might be associated with the recurrence of IgAN [53, 54]. Park et al. [22] have demonstrated that basiliximab therapy has no effect on the recurrence of IgAN after renal transplantation. This was in contrast to our findings that suggested a reduced risk of recurrent IgAN after renal transplantation with basiliximab treatment. Previous studies showed that complement activation was involved in the occurrence and development of IgAN [55, 56]. Thus, basiliximab might reduce the recurrence of IgAN by inhibiting complement activation and deposition by inhibiting T cell-mediated cellular rejection.

Tacrolimus (FK506) is a calcineurin inhibitor, often used as maintenance immunosuppression therapy after kidney transplantation [57]. Nevertheless, the protective effect of tacrolimus against recurrent IgAN after renal transplantation remained controversial. Ortiz et al. found no difference in tacrolimus use between the patients with recurrent IgAN and those without recurrent IgAN [21]. Conversely, Lionaki et al. [25] suggested that tacrolimus use might be linked to the lower rate of recurrent IgAN. While our meta-analysis revealed that tacrolimus might prevent recurrent IgAN, the result remained unstable. The specific molecular mechanism occurs via K506 binding to FK506 binding protein 12 in lymphocytes, forming a complex that binds specifically to calcineurin, inhibiting its activity. This blocks the dephosphorylation process necessary for gene expression in early lymphocytes, which in turn inhibits the activation of T cell-specific transcription factors (NF-AT) and the synthesis of interleukins. Tacrolimus further inhibits the proliferative response of T and B lymphocytes, the production of cytotoxic T cells, and the ability of T cell-dependent B cells to produce immunoglobulins [58]. Additionally, Kim et al. [59] concluded that tacrolimus use was associated with a decrease in total serum IgA1 concentration after renal transplantation. Studies confirmed that IgA deposits in the mesangial area in IgAN were mainly of the IgA1 subtype and not the IgA2 subtype [60]. These might be related to the use of tacrolimus in reducing the recurrence of IgAN.

Finally, there were some limitations in our study. As for some influencing factors, there were a small number of retrieved articles with small sample sizes. Moreover, the research results might have been affected by the quality of the original research, which showed certain bias in our analysis. Our meta-analysis could not take into account the interrelationship of factors as multivariate analysis. We further propose that studies might establish long-term follow-up periods to draw more comprehensive and objective conclusions.

## Conclusion

In general, tacrolimus and basiliximab use were protective factors against recurrent IgAN after renal transplantation, whereas donor age, patient age at transplantation, time from diagnosis to ESRD, previous transplantation, living donor, related donor, proteinuria, and serum IgA level were risk factors for recurrent IgAN after renal transplantation. Donor age, time from diagnosis to ESRD, previous transplantation, and tacrolimus use should be interpreted with caution. Clinical decision making should warrant further consideration of these risk factors and further research is still needed, including studies with large patient samples, and multi-centered and high-quality randomized double-blind controlled trials.

## Supplemental Data

Supplementary data are available at the following link: https://www.bjbms.org/ojs/index.php/bjbms/article/view/8369/2660.
